# Supplementary material for: Plant tissue analysis as a tool for predicting fertiliser needs for low cyanogenic glucoside levels in cassava roots: An assessment of its possible use
Source: PLoS One. 2020 Feb 13;15(2):e0228641. doi: 10.1371/journal.pone.0228641 (PMC7018020; doi:10.1371/journal.pone.0228641)
Supplement: S1 Text — (DOCX) [file pone.0228641.s003.docx]

**Soil profile description**

Profile number: P-NAL Date: 2015/05/02

Region: Mtwara District: Mtwara Area: Mikindani

Location: Naliendele Agricultural Research Institute, Cassava research fields

Coordinates: S 10°22'58", E 040°10'01" Elevation: 145 m

Season/Weather conditions: End of rainy season

Soil Name: Classification FAO: Ferrosol Soil Taxonomy: Oxisol

Soil moisture regime: Ustic

Soil temperature regime: Isohyperthermic

Landform: Dissected Plateaux

Macro relief: Upper slope on ridge

Micro relief: None

Parent material: Quaternary, Neogene, Jurrasic and Cretaceous sediment

Geological formation: Developed on Quaternary, Neogene, Jurrasic and Cretaceous sediment

Site characteristics: Slope gradient: 0 - 2%

Slope type of: very gently sloping

Length of slope: 75m

Position on slope: Lower slope

Natural vegetation: Shrubs: 15%, Herbs: 20%, Grasses: 60%, Bare ground: 5%

Land use: Rain fed arable cultivation of cassava as a mono-crop; land now under fallow but still has some cassava outcrops interspersed between grass, shrubs and herbs.

Surface characteristics: Rock outcrops: None; Surface stoniness: None; Sealing/crusting: None; Erosion: by water; Type: Sheet and rill erosion; Degree: Slight; Deposition: Not evident; Natural drainage class: Well drained.

Ap 0 - 12 (7 - 16) cm; dark brown (5YR 3/4) moist. Sand. Very friable moist, non-sticky and non-plastic wet. Weak medium crumbs. Common very fine pores. Many very fine roots; clear wavy boundary.

Bs1 16 - 48 (43 - 53) cm; red (5YR 3/6) moist. Loamy sand. Very friable moist; non-sticky and non-plastic wet. Weak medium sub-angular blocks. Common very fine pores. Many very fine roots; gradual smooth boundary.

Bs2 48 (43 - 53) - 104 (84 – 124) cm: red (5YR 4/6) moist. Sandy clay loam. Friable moist, slightly sticky and slightly plastic wet. Weak to moderate medium sub-angular blocks. Common very fine pores. Common very fine roots, few open burrows; diffuse smooth boundary.

Bs3 104 (84 – 124) - 160+ cm: red (5YR 4/8) moist. Sandy clay loam. Friable moist, slightly sticky and slightly plastic wet. Moderate medium sub-angular blocks. Common very fine pores and very few medium roots. Common very fine roots.

**Analytical data for soil profile**

| Parameter | Horizon | | | |
| --- | --- | --- | --- | --- |
|  | Ap | Bs1 | Bs2 | Bs3 |
| Depth (cm) | 0 – 12 | 12 – 48 | 48 – 104 | 104 – 160 + |
| Clay % | 12.26 | 15.75 | 14.00 | 11.26 |
| Silt % | 2.37 | 3.37 | 1.13 | 2.87 |
| Sand % | 85.37 | 80.88 | 84.87 | 85.87 |
| Texture class | Sa | SaL | SaCL | SaCL |
| Silt/clay ratio | 0.00 | 0.05 | 0.00 | 0.00 |
| Bulk density g/cm^3^ | 1.54 | 1.50 | 1.43 | 1.34 |
| pH H_2_O 1:1 | 5.2 | 5.0 | 4.8 | 5.0 |
| pH KCl 1:1 | 4.1 | 3.8 | 3.7 | 3.7 |
| pH H_2_O 1:2.5 | 5.5 | 5.4 | 4.8 | 5.0 |
| pH KCl 1:2.5 | 4.2 | 4.0 | 3.8 | 3.9 |
| NaF | 7.7 | 8.0 | 8.3 | 8.7 |
| Organic C% | 0.20 | 0.20 | 0.15 | 0.10 |
| Total N% | 0.03 | 0.03 | 0.03 | 0.03 |
| C/N | 6.67 | 6.67 | 5.00 | 3.33 |
| Avail. P mg/kg | 0.48 | 0.20 | trace | trace |
| CEC (1 *M* NH_4_OAc cmol/kg) | 8.6 | 3.6 | 3.0 | 3.6 |
| Exch. Ca cmol/kg | 0.31 | 1.10 | 0.31 | 0.05 |
| Exch. Mg cmol/kg | 0.15 | 0.10 | 0.04 | 0.85 |
| Exch. K cmol/kg | 0.05 | 0.07 | 0.16 | 0.15 |
| Exch. Na cmol/kg | 0.05 | 0.03 | 0.02 | 0.02 |
| Total Reserve Bases cmol/kg | 0.56 | 1.3 | 0.53 | 1.07 |
| Base saturation % | 6.5 | 36.1 | 17.7 | 29.7 |
| Exch. Al (cmol/kg) | 0.08 | 0.22 | 0.40 | 0.36 |
| Exch. H (cmol/kg) | 0.09 | 0.19 | 0.17 | 0.23 |
| ECEC cmol/kg | 0.73 | 1.71 | 1.10 | 1.66 |
| Fe mg/kg | 16.02 | 10.86 | 4.72 | 2.01 |
| Cu mg/kg | 0.19 | 0.19 | 0.05 | 0.19 |
| Zn mg/kg | 0.18 | 0.10 | trace | trace |
| Mn mg/kg | 12.68 | 9.22 | 4.92 | 2.01 |
