# Supplementary material for: Plant tissue analysis as a tool for predicting fertiliser needs for low cyanogenic glucoside levels in cassava roots: An assessment of its possible use
Source: PLoS One. 2020 Feb 13;15(2):e0228641. doi: 10.1371/journal.pone.0228641 (PMC7018020; doi:10.1371/journal.pone.0228641)
Supplement: Attachment — Submitted filename: Response to reviewers.docx [file pone.0228641.s004.docx]

Sokoine University of Agriculture

Department of Soils and Geological Sciences

P.O. Box 3008

Morogoro

Tanzania

26^th^ November, 2019

The Academic Editor

PLOS ONE

San Francisco

United States

Dear Dr Pagliari,

**RESPONSE TO REVIEWERS (PONE-D-19-21677)**

We firstly thank you for concluding that the work has some merit. The reviewer’s comments were very insightful and helpful. Thank you for helping us to present the work with greater clarity. As requested we have made changes to the work to make it fully meet the journal requirements. The changes are outlined in the Table on the next page:

PLEASE SEE THE TABLE ON THE NEXT PAGE

We hope the changes made are as required. Thank you for your patience and support.

Yours faithfully,

Matema L.E. Imakumbili.

| REVIEWERS COMMENTS | AUTHORS RESPONSES |
| --- | --- |
| 1. The paper should be reviewed to verify and correct some typos or repetition of information | The paper was edited accordingly |
| 2. Table 2 be deleted | Table 2 has been deleted, and its contents converted to text |
| 3. Tables and figures be inserted as close as possible in the first citation referring to them | This has been done, wherever possible |
| 4. Table 3 presents the summary of the analysis of variance containing: degrees of freedom, sum of the mean square for the treatments, residues and coefficient of variation | Complete ANOVA tables have now been included |
| 5. Change of title to focus on diagnosis or predicting, as nothing was done on correcting diagnosis results | The title has been changed following the advice given. It now reads as: *Plant tissue analysis as a tool for predicting fertiliser needs for low cyanogenic glucoside levels in cassava roots: An assessment of its possible use*. The objectives have also been adjusted accordingly. |
| 6. Wrong use of the terms 'balanced NPK fertilizers. Authors to consider to relate their findings to the level of nutrient use efficiency of the varieties used. | The word balanced has been removed to avoid confusion. We meant to say N, P and K supplied together. This has been clarified in the text.  We have decided not to get into agronomic efficiencies or nutrient use efficiencies and their relation to balanced nutrition, as this is not the objective of the study. |
| 7. Add to discussion the actions to undertake to reduce HCN content of the roots or leafs after doing plant tissue analysis. | This has been added, but only for the second experiment. it was more applicable there. Lines 648 – 651, 665 - 667 |
| 8. How do the results/conclusions of this study relate to roots HCN content at 10-12 months after planting and 15 months after planting? Given the fact the longer the cassava stays in the field, the higher the root yield, will the HCN content reduce with time? | This has now been included to enrich the text.  Lines 360 – 361, 698 |
| 9. Statistical analysis done on yearly basis is justified due to the fact that the soil has been corrected for S, Zn and Cu in the second year. Thus, merge Tables 5 and 6 and show results for each year for all the varieties where applicable. | Table 5 has been deleted and its contents inserted as text. Combining the Tables is not possible as they contain different information although similar. |
| 10. Lines 237-255: indicate plant age for all the analyses for both pot and field experiments | Plant ages have been now included and some corrections were made to the precise planting and harvesting times |
| 11. Lines 317-318: Revise the conversion of 0.54 and 0.51mg/100g. It gives 5.4 and 5.1mg/kg instead of 54 and 51mg/kg | The changes have been made |
| 12. Line 416: Is it not Table 5 instead of Table 6? | No its Table 6, but since Table 5 was removed it has now become Table 5. |
| 13. Table 7 shows contradictory results, which prevent any generalization of the conclusions of this study | The contradiction has been mentioned, but with the changes made to the objectives, we think the generalisation can still stand. |
